# Red Bull PET/CT

**DOI:** 10.1055/a-2195-0138

**Published:** 2023-12-22

**Authors:** Sebastian E Serfling, Andreas Buck, Steven P Rowe, Takahiro Higuchi, Rudolf Werner

**Affiliations:** 1 Department of Nuclear Medicine, University Hospital Würzburg, Würzburg, Germany; 2 Division of Molecular Imaging and Therapeutics, Department of Radiology, University of North Carolina, Chapel Hill, United States; 3 9173 Goethe University Frankfurt, University Hospital, Department of Nuclear Medicine, Clinic for Diagnostic and Interventional Radiology and Nuclear Medicine, Germany

**Keywords:** Red Bull, Energy Drink, taurine, [18F]FDG

## Abstract

We report on a patient diagnosed with Hodgkin Lymphoma who was scheduled for [ ^18^ F]FDG PET/CT as part of routine follow-up after treatment with two cycles of chemotherapy and mediastinal external beam radiation. Although the patient was advised to fast for at least four hours, an energy drink (Red Bull ) was ingested right after radiotracer administration, which led to increased uptake in the large skeletal muscles, thereby rendering this scan as non-diagnostic. After strictly following respective dietary recommendations, the repeated scan then provided excellent image quality and revealed response to treatment. In the present case report, we discuss the impact of major ingredients (sugar, caffeine, taurine, glucuronolactone) of Red Bull on large muscle uptake, which may also apply to “sugar-free” types of this popular energy drink. Moreover, this case reports demonstrates the importance to inform patients that they should avoid intake of energy drinks not only prior to but also after injection of [ ^18^ F]FDG.

## Introduction

Current guidelines recommend to avoid intake of coffee prior to injection of [ ^18^ F]fluorodeoxyglucose ([ ^18^ F]FDG), as this may hamper uptake in sites of disease and also increase physiological biodistribution, thereby limiting diagnostic accuracy 1 . Red Bull is nowadays one of the most popular soft drinks with the highest rates of brand usage and brand awareness among all generations 2 . In the present case, we report on an unintentional ingestion of this energy drink right after injection of the radiopharmaceutical, thereby emphasizing the need to inform patients about avoiding intake of these types of beverages not only prior to, but also after tracer injection.

## Case Report

**Fig. 1:**
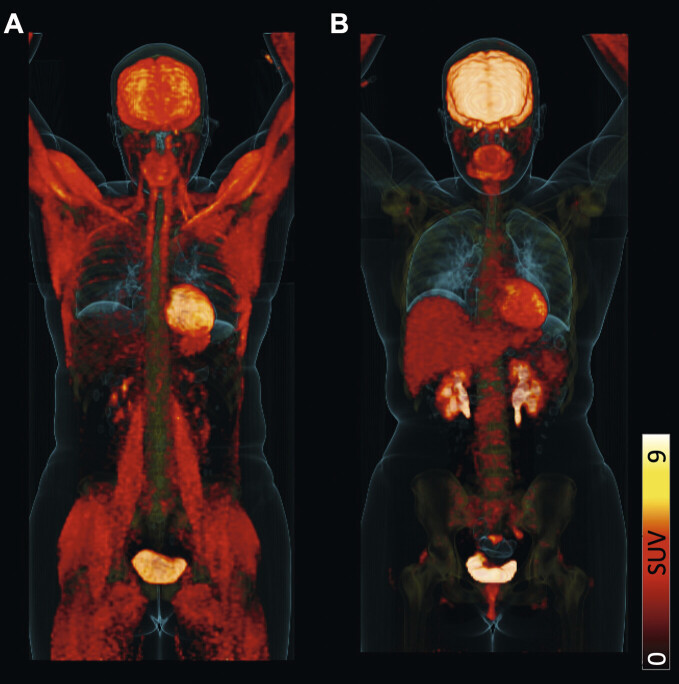
Patient with Hodgkin Lymphoma who was scheduled for [18F]FDG PET/CT. In ( **A** ), increased uptake in large muscles along with reduced brain, increased myocardial and almost absent liver uptake was revealed, thereby questioning adherence to the fasting period. The patient then reported on ingestion of an energy drink (Red Bull) right after radiotracer administration. After having strictly followed respective dietary recommendations, the scan was repeated ( **B** ), which showed normal [18F]FDG biodistribution and response to treatment.

We herein report on a patient with Hodgkin Lymphoma who was scheduled for [ ^18^ F]FDG PET/CT as part of routine follow-up after treatment with two cycles of chemotherapy and mediastinal external beam radiation. Following current guidelines 1 , the patient was advised to stop any sugar or caffeine intake at least 4h prior to molecular imaging. A blood glucose level of 97 mg/dl was recorded upon arrival at our PET/CT center. As shown on [ ^18^ F]FDG maximum intensity projection in ( **A** ) of Fig. 1 , increased uptake in large muscles along with reduced brain, increased myocardial and almost absent liver uptake was revealed, thereby questioning adherence to the fasting period. The patient then reported on ingestion of an energy drink (Red Bull ) right after radiotracer administration. After having strictly followed respective dietary recommendations, the scan was repeated ( Fig. 1 **B** ), which showed normal [ ^18^ F]FDG biodistribution and response to treatment.

## Discussion

As one of the most popular energy drinks 2 , a 250ml can of Red Bull consists of water, sugar (12g/100ml), caffeine (80mg), 2-aminoethanesulfonic acid (taurine, 1000mg) and glucuronolactone (600mg) 3 4 and has been advocated to increase (an)aerobic exercise performance 5 . Given its rather low amount of sugar relative to other energy drinks 6 , one may speculate whether additional ingredients may have contributed to the high uptake in skeletal muscles observed in the present case report. For instance, taurine can be involved in muscle contraction by increasing sarcoplasmic reticulum calcium aggregation and release 7 . As muscle fibers adjust their contractility by regulating intracellular levels of taurine 4 7 , elevated blood concentration of this amino acid after Red Bull intake may also explain increased accumulation of [ ^18^ F]FDG in the muscles. Nonetheless, it remains unclear how fast taurine is absorbed from the digestive tract or incorporated in muscular cells 4 . As such, beyond potential additive effects of taurine combined with caffeine, the observed high skeletal muscle uptake could also be predominantly explained by caffeine alone 4 . The latter ingredient can lead to increased exercise endurance by preservation of muscle glycogen and neuroendocrine activation 8 9 and thus, current guidelines also recommend refraining from intake of coffee prior to [ ^18^ F]FDG PET 1 . Taken together, beyond sugar, the excitatory effects caused by taurine, its precursor glucuronolactone, and caffeine may have all contributed to the observed increased uptake in the muscles. Thus, patients should be advised to avoid such beverages not only prior to, but also right after [ ^18^ F]FDG injection, even if labeled as “sugar-free”, as those drinks may still include the afore-mentioned ingredients potentially causing increased muscular radiotracer accumulation.
